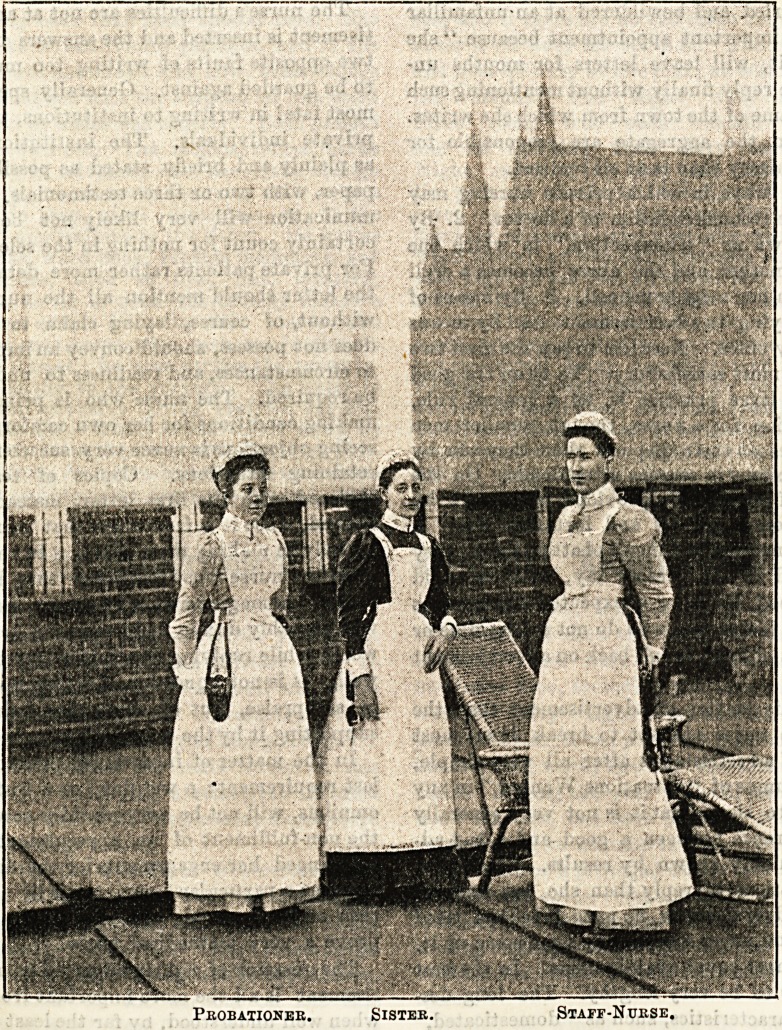# The Hospital Nursing Supplement

**Published:** 1894-12-08

**Authors:** 


### "The Hospital" Nursing Mirror.

Being the Extra Nursing Supplement of "The Hospital" Newspaper.

[Contributions for this Supplement should be addressed to the Editor, The Hospital, 428, Strand, London, W.C., and should have the word "Nursing" plainly written in left-hand top corner of the envelope.]

### News from the Nursing World.

### OUR CHRISTMAS COMPETITION.

Applications for a share in the parcels sent in for our Christmas competitions are specially urgent this year. Matrons and charge nurses have much to say about the existing need for "a little help," which, according to the old saying "is worth a deal of pity." Hospital patients *must* have Christmas gifts, there do not seem to be two opinions on this matter, and when the public is ungenerous the pockets of hospital workers are sorely taxed. It does not seem fair that those who give time, strength, and skill should feel obliged to make up for the lack of charity of the majority who cannot offer personal service. The supply of deficiencies is one of the objects of our needlework competition, and we trust that many a sick man and woman on Christmas Day will have cause to gratefully remember readers of The Hospital. All parcels should be delivered by December 18th, and should be addressed to "Nursing," care of the Editor of The Hospital, 428, Strand, London. The name and full address of the sender should be placed in each, and everything received will be acknowledged in the next issue of the paper.

### WORK FOR COMPETENT WOMEN.

Three ladies are announced as candidates at the approaching election of Poor Law Guardians in Liverpool. Although women have for some time past done useful work on the Boards at Toxteth Park and West Derby, the parish of Liverpool has not yet had a lady guardian. The ratepayers however, acknowledge that a great many matters connected with workhouses and infirmaries are essentially within the province of women, and with which they should be especially fitted to deal. In face of this encouragement we again urge upon our readers the necessity for those who have leisure for this kind of work preparing themselves to do it well. No woman can be useful as a guardian or inspector unless she knows exactly what is needed to ensure success in institutions; housekeeping, sanitation, hygiene should all be familiar to her. A thorough knowledge of the administration of sick wards is desirable, and it cannot be grasped haphazard, it must be studied intelligently. With this knowledge, added to trained powers of observation, a warm interest in humanity, and unlimited tact (without which all else is useless), the women guardians and inspectors of the future should prove valuable auxiliaries.

### THE WORK OF THE CHILDREN.

"None of them seem very ill," remarked one of the visitors, who passed out of the bright wards into the foggy atmosphere of Cheyne Walk last Tuesday, the "Incurable Children's" show of work on that and the following day having attracted many guests to Chelsea. Perhaps not "very" ill, in the conventional meaning of the word, but they are all chronic invalids at this children's hospital, in spite of the cheerful voices and merry laughter. "Spines" or "hips" are the children's own brief diagnosis of their cases. Nearly all were in bed, lying flat and still, under the restoring influences of modern surgical and nursing appliances. The needlework was marvellously fine, both in colour and artistic design, and it was difficult to believe that the feeble fingers of little patients could become so skilful. Great credit is due to the patient teachers, whose pupils accomplish such excellent results. The articles displayed (not for sale) with justifiable pride at this annual exhibition were greatly admired. The condition of the children and the wards being alike excellent, the visitors took away pleasant impressions of the pretty riverside institution.

### PROGRESS AT DURHAM.

It is reported that the professors and lecturers at Durham desire that University to be empowered to grant degrees to women in arts, medicine, and science. Dr. Kitchin, the Dean of Durham, and Warden of the University, has called Convocation together, on December 11th, when it is proposed to affix the University Seal "to a petition to Her Majesty the Queen for a supplementary Charter to enable the University to confer degrees upon women."

### A CONVALESCENT HOME.

At a recent meeting of the members of the Council of the Stockton and Thornaby District Nursing Association, at which the Marchioness of Londonderry presided, the Convalescent Home at Middleton St. George was specially mentioned, and the necessity for its establishment on a firm basis was strongly urged. The lines on which it has been started are considered satisfactory, and the value of such an institution being acknowledged, the co-operation of working men is to be invited to convert Middleton St. George into a permanent instead of a temporary home.

### A PRACTICAL ILLUSTRATION.

A new fever hospital is to be built near Kingstown, a satisfactory site having been already secured. If evidence were wanting that such a hospital is badly needed, it was supplied while the inquiry as to compensation, &c., was in progress. A poor woman, in the early stage of small-pox, presented herself at the Town Hall to know where she was to go. The commissioners had no alternative but to send her in an ambulance to Cork Street Fever Hospital, Dublin, a distance of nearly ten miles.

### QUEEN'S NURSES IN IRELAND.

The Queen's nurses are evidently welcomed and valued in Ireland, and some of them carry on their work in strangely out-of-the-way spots. One of the most remote districts is "The Rosses," in Co. Donegal, some thirty-five miles from a railway station. An outside car conveys the General Superintendent to Burtonport, and from there she crosses the Sound in an open boat to the island. The country of The Rosses is wonderfully barren, but it has a population of 10,000. "Very strange, yet pleasant," writes the Superintendent, Miss Dunn, "it was to see in this little cabin on the heights of Aranmore, the Queen's nurse moving about in her neat uniform, reducing to nursing order the mud-floored room, while the poor patient comprehended for the first time in his life what skilled nursing meant." It will give some idea of the difficulties of district nursing to hear that this patient, who had typhoid, and a temperature of 104 degrees, was found lying under four blankets and a heavy quilt, and wearing two suits of clothes! The Queen's nurse must have had no easy task, yet she did her duty with such tact that the patient's mother accurately carried out all her directions, and during Dan's convalescence she often expressed compunction for "having nearly kilt him with clothes." Miss Dunn and her nurses are to be congratulated on their adaptability to circumstances, and it is small wonder that their services are so highly prized by the sick poor in Ireland.

### PREPARATION FOR A USEFUL LIFE.

The practical education of French girls already includes departments which might advantageously be introduced into other countries. In Paris there are at present two higher grade schools in which girls can continue the studies entered upon in the primary schools, whilst they receive in addition excellent instruction in housework, needlework, cooking, marketing, hygiene and thrift. They can also be taught drawing, painting, artificial flower making, book-keeping, besides modern languages. The needlework department includes fine embroidery and dress-making, and every branch of instruction is placed in the hands of competent teachers. Not only do schools of this kind exist for the lower classes, but the same kind are provided for the wealthy, who stand in equal need of training in the administrative duties which lie before them. Technical schools for those who have to make their own living are also said to be equally successful in France. Aspirants to the profession of nursing whilst waiting till they are old enough to enter on training, might fill up their time profitably in such institutions.

### NEW YORK ALUMNÆ.

The "Alumnæ House" for nurses who have graduated at the New York City Training School is situated in West Twenty-first Street, and is a pleasant and popular establishment. It is ably conducted by Miss U. Z. Foote, herself a member of the Alumnæ Association, Mrs. F. M. Fenton being the president, and the officers well-known nurses. There is a sick benefit fund connected with the association, which is already properly valued, and a monthly social meeting, at which a lecture is given, takes place at the house. In November the subject taken was "Nursing in Cases of Ear Disease," by Dr. George B. McAuliffe. The association numbers 115 members.

### OUR AMERICAN SISTERS.

Fortnightly meetings are held in connection with the Johns Hopkins Hospital Training School Journal Club. A head nurse presides over each meeting, and chooses the subject for the evening. These are generally selected from articles in current nursing literature, as the Journal Club aims at keeping members of the school informed as to progress abroad and at home. It also helps nurses to acquire facility and confidence in speaking and reading aloud. Amongst the subjects named for discussion are District Work, the Rest Cure, Nursing of the Insane, Private Duty, Nursing in Foreign Countries, &c.

### SISTERS IN WESTERN AUSTRALIA.

Under the title of "Sisters of the People," three nurses have recently been inducted into the Perth Central Mission, Australia. Sister Alice, trained at the Adelaide Hospital, and will work with Sisters Marian and Louis at the Perth headquarters; Sister Hannah, trained at the Adelaide Children's Hospital and in district work, is to be settled at Geraldton, and the third, Sister Agnes, is to live at Freemantle. The service of dedication took place at Perth in October.

### SHORT ITEMS.

A course of lectures on sick nursing is now being delivered, under the auspices of the Wigan Mining and Technical School, by Miss Peele.—*Nursing Notes* for December contains much interesting matter; it is the journal of the Workhouse Infirmary Nursing Association and the Midwives' Institute.—The fifth report of the Glasgow Sick Poor Nursing Association shows a satisfactory record of work amongst the poor, for whom it provides night as well as day nurses; thirty-eight private nurses are also on the books of this association.—The claims of the Sleaford District Nurse Fund have been set forth in various local places of worship, the collections at the parish church amounting to £9 6s.—The first annual meeting of the Lincolnshire Nursing Association was recently held, under the presidency of the Earl of Winchilsea.—A woman student was returned as a member of the Students' Representative Council of the University of Edinburgh.—Mr. Bonham-Carter, Chairman, and Mr. Walter Richmond, Hon. Secretary of the Paddington and Marylebone District Nursing Association, are appealing for new subscriptions to obviate the need of reducing the staff. Donations will be gratefully received by Miss K. Pearce, Nurses' Home, 510, Edgware Road.—A most enjoyable concert, attended by all the patients not confined to their beds, and a large number of nurses, was given last week at the Royal Hospital for Incurables, Donnybrook. The men's day-room was very prettily decorated for the occasion, and every item of an excellent programme was thoroughly appreciated.—A silver medal for five years' faithful work on the staff of the Blackheath and Richmond Institutions has been awarded to Nurse Elena.—A successful concert was recently given at Shaw in aid of the Crompton Sick Nursing Association.—A sale of work on behalf of the funds of the York Home for Nurses lately took place at Monkgate.—An interesting lecture on "Child-life" was delivered by Dr. Altham to the members of the Penrith Women's Sick Nursing Association, a week or two back, and was much appreciated.—Subscriptions are solicited by the hon. treasurer of the Torquay Nurses' Institute, affiliated with the Queen's Jubilee Institute; the six nurses employed in district work find their labours much appreciated.—The annual meeting of the Skipton Nursing Association took place in the end of November; the two trained nurses have paid 2,889 visits during the year.

### The Dietetic Management of Infancy.

By George Dickson, M.B., C.M.

### I.—INTRODUCTORY.

Perhaps there is no department of medical work in which the doctor and nurse have to do battle with so much popular ignorance and popular prejudice as in the dietetic management of infants and children.

The fundamental principle on which is founded all rational practice in the world of medicine, and which is of especial importance in this department, is that the methods of nature are the best, and that artificial methods when necessary are only likely to be satisfactory in so far as they approximate to those which nature herself employs. Common sense as this principle may seem, there is nothing in practice more astonishing than the frequency with which we find it absolutely ignored by parents with results the most disastrous. It is no exaggeration to say that improper feeding causes the death every year of thousands of children, and blights the happiness and cripples the whole after usefulness of thousands more.

### General Management.

There are three things apart from diet which, while of importance to all, are matters of paramount necessity in childhood. These are fresh air, cleanliness, warmth. Every child after the first two or three weeks of life should be well wrapped up, and daily exercised in the open air unless the weather is absolutely prohibitory. It should be bathed twice daily with tepid water, the temperature being gradually reduced as it grows older and hardier. It should be lightly, loosely, and warmly clad. The practice of pinning a child tightly up in his clothes is a bad one, hampering as it does the movements of the chest, and so preventing full expansion of the lungs. As regards material, undoubtedly the best is flannel, but flannelette is cheaper, and is the next best substitute. Not the least important part of the clothing is the binder. This should consist of a strip of flannel, wound twice round the body, and pinned somewhat tightly round the pelvis—not so tightly round the abdomen—to prevent its slipping up. It is said to be more elastic if cut diagonally. (Eustace Smith.)

### Breast Feeding.

In the dietary nature's method is infinitely the best, and every mother should, for a time at least, endeavour to suckle her child. The secretion of milk does not become fully established till the third day after delivery, but before this there is present in the breasts a secretion rich in fatty particles—the colostrum. It is a good practice to put the child to the breast shortly after labour is over, and to keep applying it at intervals till lactation fairly commences. The child in this way gets the benefit of the above-mentioned colostrum, which acts as a gentle natural aperient, and clears its bowels of the meconium with which they are loaded. The nipple also becomes well drawn out before the breast gets distended and hard. Perhaps the most important reason is that the child at the breast stimulates the uterus to contract and hastens the involution of that organ. It is for this reason that, in cases where post-partem hæmorrhage is dreaded, putting the child to the breast immediately after labour becomes a valuable preventive means of treatment. The practice of giving sugar and water, &c., to infants until the milk appears should always be condemned as being an ignorant attempt to improve upon nature's methods. When the secretion is fully established the infant should be put to the breast every two hours during the day for the first six weeks. It should be fed about ten or eleven at night and not again till five or six next morning. If these intervals are strictly adhered to at the first the child soon gets into regular habits, and much annoyance and many sleepless nights are avoided to all concerned.

The mother should regulate the flow of milk by keeping a finger on either side of the nipple while the child is sucking. If allowed to flow too freely, or the child allowed to swallow it too greedily, the milk will almost certainly be rejected again by vomiting. After each application the nipple should be well washed with tepid water and dried with a soft cloth. By this means abrasions, &c., will generally be avoided. When the child is six weeks old the intervals should be extended to three or even four hours.

Provided the child is thriving, nothing but the breast ought to be given until it is seven or eight months old. It is a matter of common observation that many children thrive well on the breast alone till twelve months or even more, but it is better about the seventh or eighth month to accustom them to other food, and at this age it may be safely be given.

Earlier than seven months the salivary glands and pancreas, whose function it is to digest starchy food, have not begun to secrete their juices in sufficient quantity, and the practice of giving cornflour, arrowroot, &c., before this age is perhaps the most common cause of the manifold digestive disorders of infancy. It may be laid down as an axiom that starchy food, unless predigested, should never under any circumstances be given to children under seven months, and provided the child is thriving there is no occasion whatever to add even predigested food to his dietary before this period.

At or about the seventh month a teacupful of boiled cow's milk should take the place of the breast meals twice daily. If it disagree it should be diluted to one half or less with water to begin with. When the stomach has became accustomed to the cow's milk entire wheaten flour is the best addition. To prepare this the wheaten-flour should be tied up in a pudding cloth and boiled for three hours to digest it thoroughly. The outer soft part of the mass should be scraped away and the inner and harder part grated to form the food. Of this a teaspoonful, rubbed up first with a little cold milk, should be added to the cow's milk meal, at first once, and then twice daily, but never oftener. In a smilar manner a teaspoonful of Nestlé's or Mellin's food, the latter being most convenient, may be given instead of, or alternately with the flour. Both are malted foods. Benger's food agrees well with babies, but it is a pancreatised food and, like all pancreatised and peptonised preparations, its use for any great length of time tends to weaken the stomach. The child may now have a rusk occasionally, and the yolk of an egg beat up with a little milk and sugar forms a useful addition. The diet may be varied by giving occasionally a meal of about six ounces of mutton or chicken broth, or beef-tea, or a little well-made oatmeal porridge. Beef-tea for children should only be half the strength of that for adults, *i.e.*, half a pound to a pint. The amount of fat in the diet is well increased by a tablespoonful of cream twice daily.

### Where to Go.

Trained Nurses' Club, 12, Buckingham Street, Strand.—On December 14th, at 7.45, Dr. Tom Robinson will lecture on "The Impressionable Temperament."—Preparation class for L.O.S. examination in April will begin early in January. Particulars can be obtained from the secretary at the club. —Nursing Talks. On December 11th the subject will be "Cookery for Children." Ticket for single lecture (which begins at 3 p.m.) 1s. 6d.

Albert Hall.—Organ Recitals every Sunday afternoon, admission free.

Albert Hall.—*Truth's* Toy Show on December 19th and 20th.

Westminster Town Hall.—On December 20th the annual sale of work made by the members of the Children's Salon will be opened by Princess Edward of Saxe-Weimar. The proceeds will be devoted to the completion of the endowment of a cot at the North-West London Hospital.

### Nursing in Military Hospitals.

### II.—DAY AND NIGHT DUTY.

In military wards the sister comes on duty later than the ward orderly, who holds much the position of a staff nurse. He, aided by the convalescents (if there are any in the ward), fetches the coal for the day, makes those beds where no assistance is required, gives water to the patients who wash in bed, sweeps and dusts the wards, and does all emptyings. When the sister enters she is greeted with a pleasant good morning by orderly and patients. She takes temperature, pulse, respiration, gives medicines, and inquires of each patient how he is. She reads the report of the night orderlies, and enters all particulars on the charts, giving any stimulants or nourishment due, then she usually has bad patients to wash, and is helped by the orderly in making beds that need special care. She usually washes the backs of bed-ridden patients, and if the orderly is new to the ward she shows him how these things should be done. At about ten a.m. the surgeon of the ward goes round, accompanied by sister and orderly. Each patient who is up stands by his own cot. The medical officer marks up each man's diet a day in advance, and the diets, though each comes up separately, are on much the same scale as in a good civil hospital. He then gives directions as to the nursing, dressings, and applications to the sister. He writes fresh prescriptions in the ward book, repeats medicines, and orders necessary supplies, such as tow, lint, mustard, bandages, &c. As soon as he is gone the orderly makes a list of all the diets and extras from the diet boards, and takes them down to the cook-house with each man's diet tin or basin needed for his dinner; he also carries the prescription book and empty bottles to the surgery. The sister in the meantime has been probably giving beef-tea, egg-flip, stimulants, &c., that are due. Then, unless called away by drill or parade, the orderly helps the sister with dressings, poultices, rubbings, inhalations, &c.; then he brings up stimulants which she measures, lemons to make lemonade for bad cases, beef-tea, meat for special cases, and eggs. Whilst making these special extras the sister usually holds a class of instruction for the orderlies from all her wards. New "admissions" come, and must be got to bed and attended to; "before food" medicines must be given. The orderly fetches the dinners, the sister sees to them for all bed cases, and at one p.m. she leaves for her own dinner. The orderly gets his at the same hour, but if there are cases that cannot be left some arrangement is made. At two p.m. the sister returns to her patients, gives medicines, stimulants, changes poultices, tho orderly being probably busy giving in kits, or he may be taken for a lecture or parade. At three he has to fetch up cook-house extras, such as milk, beef-tea, lemonade, and puddings, after which there is usually a quiet time till tea, and if there are no bad cases requiring her attention the sister is off duty till six p.m. The orderly gets up the teas and goes off duty at five p.m., at which hour the night orderlies mount. The night duty is usually done by three men, who each remain on for four hours, and on finishing their duty leave a written report. Night specials, as they are called, are only detailed for patients unsafe to be left, such as delirious or enteric cases, or operations. At other times the duty is done by corridor orderlies, who visit the wards at stated times. A ward master or non-commissioned officer also mounts duty each night to see that all orders are duly carried out, and the men in their appointed posts. Where no night orderly is mounted in her wards the sister does the whole evening's work, giving medicines, nourishment, stimulants, taking temperatures, doing dressings, making beds, and the usual routine of straightening up for the night. She writes all orders for medicines, stimulants, and poultices, patients to be visited, observations to be made, clearly in a book, and the ward master for the night is responsible for the carrying out of these orders. Such is a rough sketch of work as carried out in a military hospital.

### The Government Civil Hospital, Hong Kong.

### I.—THE HOSPITAL AND COMPOUND.

Having but a limited time at my disposal before joining the homeward-bound mail, and recollecting a promise made to friends interested in hospital work, I hastened off to the western part of the town to find the Government Civil Hospital, and to study European nursing in Hong Kong.

The hospital is situated above the main street of this densely populated colony. Occupying a somewhat elevated position, it escapes a good deal of the unpleasant atmosphere which surrounds the dwellings below.

Originally instituted for the benefit of Government servants, it is now open to all comers desiring treatment, either as interne or externe patients, and there are about one hundred and twenty beds for their accommodation. The building is not a prepossesing one in appearance, but has a pleasant outlook—on the harbour on one side and on the green slopes of the "Peak" on the other, and is provided with the usual spacious verandahs which render the houses so charming. The hospital has the reputation of being one of the coolest spots in the colony (beneath a certain elevation), an advantage only to be understood by those who have passed a summer in such a climate as Hong Kong. The compound in which it stands extends over several acres, and contains not only the hospital with its attendant block, but both the European and Chinese lunatic asylums, the staff quarters, and a small isolation hospital, used frequently for stray cases of small-pox. The ground floor consists principally of the receiving ward, dispensary, offices, storerooms, &c.; while the cook-house—a separate building—is connected with it at one extremity by means of a short covered way.

After an excursion round these premises, I was conducted to the first floor by a broad polished wood staircase and delivered over to the sister in charge who, clad in white summer uniform, terminating in a very proper little cap, volunteered to show me round the wards. She told me that formerly there were but six sisters, whilst now they numbered nine, including the matron or head sister. They were on "duty" eight hours at a time—from six a.m. to two p.m.; two p.m. to ten p.m.; ten p.m. to six a.m., two sisters for the morning work, two for the afternoon, and one for the night. During this year a block, formerly under the care of ward masters, has been partially taken over by the sisters, necessitating an increase in their number.

The matron's principal duties are the management of the linen for the entire compound, the supervision of the female lunatics, and the charge of the sisters' quarters, a palatial looking residence about four minutes' walk from the hospital, whither the sisters resort for meals unless detained in the wards.

During the winter months the white uniform is exchanged for grey cotton, and for the chilly days of early spring woollen dresses are provided. The Chinese attendants wear whiteish dresses, white stockings, and blue ribbon garters and cloth shoes, and in winter small round black silk caps, ornamented with a coloured top-knot to protect their shaven heads from the inclemency of the season. They are always supposed to wear their queue hanging straight down, for if wound round the head it is considered a mark of disrespect.

The first floor is divided into two general wards and four private rooms, the latter containing either one or two beds, as desired, forming a first or second class ward accordingly. The second floor is similar to the first, with the exception of the private wards, of which there are but two, and an additional space now being converted into a theatre, a long desired and necessary adjunct, operations formerly being conducted either in the ward or receiving-room, as happened to be convenient.

### Old Time Nurses.

(*Continued from page* lxi.)

### V.—OUT OF WORK.—(*Continued.*)

Among the causes which contribute to the difficulty experienced by many older nurses in finding work, a good proportion can undeniably be traced to want of the simplest business ability—it might even be said, of common sense—on the part of the nurses themselves. There may be something in the nurse's life which has the effect of blunting her business perceptions, so few are there who escape in later life the infection of incapacity for looking after their own interests. The woman who is prompt, capable, and keen eyed in the sick room, ready of resource and undaunted in emergency, will be flurried and bewildered at an unfamiliar journey, will break an important appointment because "she has no time" to keep it, will leave letters for months unanswered, and post the reply finally without mentioning such a little detail as the name of the town from which she writes. These little matters in the aggregate are responsible for more "out of work" misery than is at all realised.

There are four main ways in which private nursing may be obtained—1. By the recommendation of a doctor. 2. By what is commonly known as "a connection," in which one patient recommends another and the nurse becomes a well recognised personage in her neighbourhood. 3. By means of advertisement, or replying to advertisement. 4. By means of an agency or registry office. Needless to say the first two methods are by far the most satisfactory. To be on the good books of a doctor in large practice is, as a general rule, quite sufficient provision for a nurse, though smaller men have mostly more nurses at their disposal than they can by any possibility need, except in seasons of epidemic. On the whole the proportion of nurses fully provided with work by medical recommendation is a small and fortunate one. A satisfactory private connection is difficult of attainment; very slow in formation, and in the present day, with its rapid changes of neighbourhood, liable to unexpected fluctuations and failure. There are few nurses who do not at one time or another find themselves obliged to fall back on advertisement or agency office.

It is precisely in this matter of advertisement that the business faculties of the nurse are apt to break down most hopelessly. The art of advertising is after all very simple, but a glance at the columns of "Situations Wanted," in any newspaper, is enough to show that it is not very generally understood. The difference between a good and a bad advertisement is very quickly shown by results. One nurse will have more applications in reply than she can answer. Another will tell you advertising is no good, she has tried it innumerable times, and never known any good come of it. Yet both may have almost equal qualifications. In the first place it is not necessary to be very lengthy. The long descriptions of personal characteristics, such as "domesticated," "kind," "companionable," "trustworthy," "cheerful," are all beside the mark. Everyone knows how lightly these expressions are self-appropriated; no one supposes that an attendant is unkind because she does not publicly plume herself on her humanity, and the very idea of a consciously cheerful nurse suggests an endless train of reminiscences of former patients which most invalids could thankfully dispense with. The main object should of course be to convey as much information as possible about the applicant in the space available. A common mistake, especially among older nurses, is to advertise all the conditions desired, and omit the qualifications. The advertiser wishes to go abroad, or to the seaside, to have an elderly patient, or a child, to do no night work, or no lifting; but gives no particulars which can assist the employer in identifying her as the exact person required; very often a mere trifle turns the scale. The "good walker" may be gladly secured by the friends of some restless old gentleman who has worn out all his attendants; and such talents as good reading, needlework, good lifting, &c., are valuable additions to the qualifications of a nurse, and are quickly seized on as the eye travels down the column devoted to "As Nurse-Attendant." More important details of hospital training, experience in various difficult cases, and special knowledge, as of massage, &c., are less likely to be omitted, but even these are by no means always clearly put forward.

The nurse's difficulties are not at an end when the advertisement is inserted and the answers begin to come in; the two opposite faults of writing too much and too little have to be guarded against. Generally speaking the first error is most fatal in writing to institutions, the second in writing to private individuals. The institution will want the facts, as plainly and briefly stated as possible, on one side of the paper, with two or three testimonials. A long effusive communication will very likely not be read at all, and will certainly count for nothing in the selection of the candidate. For private patients rather more detail will be acceptable; the letter should mention all the nurse's qualifications, and without, of course, laying claim to knowledge which she does not possess, should convey an impression of adaptability to circumstances, and readiness to undertake whatever may be required. The nurse who is principally concerned with making conditions for her own comfort and begins with foreseeing objections is never very successful either in getting or retaining situations. Copies of testimonials should be enclosed with the first letter, instead of being promised if desired, since it is from these that many persons are guided entirely in making their choice. In replying to an advertisement the nurse must remember that her application will be received among a number of others, and that her first letter will probably decide definitely for or against her. If it is worth while replying at all, it is worth while doing it really well. It is not a question of orthography, or fine expressions, or self-praise, but of conveying definite information, and supporting it by the testimony of doctors and employers.

In the matter of interviews, punctuality is the first and last requirement; a wet day, or a lost train, or the wrong omnibus, will not be very placidly accepted as an excuse for the non-fulfilment of an appointment by a lady who has rearranged her engagements for the day in order to see the nurse at a particular hour, nor will she readily understand that an unpunctual woman may in some mysterious way prove a very methodical nurse.

The question of rightly advertising and replying to advertisement is all the more important from the fact that it is, when well understood, by far the least expensive and troublesome method of obtaining appointments. There are many good agencies no doubt, but they can seldom have more than a local connection, and the nurse may waste months in waiting for a situation in a town or neighbourhood where the nursing profession is over-stocked, while her services in another locality would be continually in demand. Moreover, the fees due to the agency when work is obtained must be taken into account. In all cases a heavy drain on the nurse's finances, they have been known in exceptional cases to amount to as much as 50 per cent. of the total earnings. We are still a long way from the admirable American system of a central registry office or bureau, to which persons in need of a nurse resort as a matter of course, and where the nurse's name and qualifications are entered on the payment of a reasonable entrance fee. The "private venture" agency in force in England, though vastly improved of late years, is still far from attracting the confidence of any very considerable number of either nurses or employers.

### Dress and Uniforms.

By a Matron and Superintendent of Nurses.

### THE WESTMINSTER HOSPITAL.

This picturesque group represents a sister, a staff nurse, and a probationer of the Westminster Hospital. Most of us are familar with the appearance of this quaint old pile, the parapets of which form so appropriate a background to the illustration. The figure standing in the centre is the sister. Her dress is made of navy blue cashmere, which is perfectly plain, and fastened in front. The skirt just clears the ground all round, and is gathered on to the bodice at the waist. A white linen apron of ample dimensions comes next, to which a square bib with straps is attached. These straps cross at the back, and fasten to the waist-band of the apron with buttons placed about three inches apart. The cap is oval in shape and composed of white spotted net, the crown being sewn into a band that fits to the head. Two rows of gophered net frilling finish the cap off round the edge, and are kept in position by a thread run through the gophers on the wrong side, which are drawn sufficiently tight to produce the required shape. Linen cuffs and collars give a neat finish to the costume at the wrists and neck.

Probationer. Sister. Staff-Nurse.

To the left of the sister stands her staff-nurse, whose dress is made of a very pretty blue and white twilled galatea, cut plain and short, but with sufficient fulness in the skirt to admit of its hanging in graceful folds from the waist. The colour is soft and pleasing in the extreme, looks clean and fresh, and washes admirably. Few materials are more suitable or becoming to a nurse than this, which in a measure accounts for its popularity. Worn over the dress is the linen apron, which, like the sister's, is furnished with a bib and straps that cross behind and fasten at the waist. The neat little spotted net cap, of which we have already given a description, is repeated in the case of both staff-nurse and probationer. Linen cuffs and collars complete this charming costume. The probationer, who is depicted standing on the right, wears the same uniform as the staff-nurse opposite. The dress, cap, apron, collar, and cuffs, are in every particular the same, and the delicate blue of the staff nurse's and probationer's dress affords an harmonious contrast with the darker shade worn by their superior, and which may be described as the special prerogative of all hospital sisters.

### Fresh Novelties in Dress Material at Messrs. Denton and Holbrook's.

Messrs. Denton and Holbrook, of Gloucester, have just brought out some attractive novelties in the way of galatea suitable for nurses' uniforms. These will prove a boon to those heads of institutions who are becoming a little tired of the prevailing monotony in blues, mauves, or reds, which form the majority of nursing costumes. Worthy of notice is a charming pale green and white striped galatea, which only requires to be known to become popular, so cool, fresh, and spring-like is its appearance. It also has the advantage of washing well, which is always a consideration. One somewhat similar in design, though less uncommon in colour, is produced in pale pink and white; it is very delicate and pretty, and could not fail to look well and prove most becoming to the wearer. Messrs. Denton and Holbrook are also showing a new material for veils. It has the appearance of gossamer, but is less expensive, and does not spoil with rain. To those nurses who are in the habit of wearing this adjunct to their bonnets we should strongly advise a trial, as it is guaranteed to give every satisfaction.

### Tweeds at Messrs. Currie, M'Dougall, and Scott.

The Galashiels tweeds manufactured by Messrs. Currie, M'Dougall, and Scott, of the Langhaugh Mills, are among the leading novelties of the season. In addition to being warm, soft, and light, their colouring is most harmonious, and the price exceedingly moderate. Shepherds and other plaids are always more or less in request, and of these there are an excellent assortment. The "Maid of Lorne" costume cloth is certain to become a favourite; also the "Mary Hamilton," which is something quite new. Those who are in want of a winter costume cannot do better than send for a catalogue.

### Notes from India.

(Communicated.)

According to the *Pioneer*, the training of female medical practitioners at the Agra Medical School cannot be said to have proved a success so far. Only seven students presented themselves for the final examination, and three of these passed in all subjects. Dr. Lukis remarks that "nearly all the female students were found to be sadly wanting in clinical and practical knowledge," adding, justly enough, that "this is a very serious matter, as mere book-learning will never enable the girls to become successful practitioners." In the general state of female education in the country it would have been unreasonable to expect the Female Medical School at Agra to be an immediate success. It is to be hoped that the results obtained next year will show improvement.

Cholera is still prevalent on the routes between Bareilly and Lucknow. The troops rather score by this, for instead of marching they go by rail between these two places.

Our Indian Medical Congress will be held in Calcutta next month. The Viceroy has consented to be present at the opening on December 24th, and their Excellencies Lord Elgin and Lady Elgin will be present at the conversazione on December 29th.

At the Congress there ought to be some interesting papers on enteric fever, for the medical officers out here have every opportunity for studying it. Probably more than 300 cases have come under my own observation during the last four years. At one time I was seeing 60 cases daily, besides convalescents. Enteric fever out here certainly differs in many ways from the same disease at home. Convalescence is always more or less interrupted by recurrences of malarial fever, and too often the sad sequel is hepatic abscess. This summer a great number of cases have been complicated by meningitis, and, a not uncommon symptom out here, paralysis of the throat. A great number have suffered from hyperpyrexia. One man, who died lately, had a temperature of 106° for four days and 105° for four more; he was only in eight days. Of course his temperature at those points was not continuous, it being reduced at intervals by phenacetin and sponging. He was sponged regularly every two hours. He had very little lung mischief and not excessive ulceration. There is a theory that enteric to a great extent is caused in this country by exposure to the sun. I am not scientific, only practical, so am shy of offering opinions in a paper like The Hospital, but an old nurse may, perhaps, offer a few remarks on what has been her experience out of a vast number of very successful cases. The less medicine used the better, and people should not be over-nursed. Tepid or cold sponging is preferable to iced water. For sordes and dry mouths, hydrochloric acid, dil. ℞ xv., aquæ ad. ℥i., every six hours, seems beneficial. Stimulants are best given only when necessary. Plenty of water to drink in small quantities at a time seems also advisable, and heads should be shaved to start with. With a persistent high temperature, cold bathing from once to three times daily is good, and if done very carefully always proves of benefit. For this, four are needed to lift and, if possible, a fifth to steady the head. A long bath is brought into the ward and placed at the foot of the bed. Two assistants stand each side and lift the patient on the sheet he is lying on (which, of course, must be a strong one), and gently move him down over the foot of the bed into the bath. A towel is rolled up for a pillow for his head, and the sheet folded round him. The bath is gradually cooled down with iced water. If the patient is started in a tepid bath the shock is not felt. A patient who is nervous should never be bathed, and I always warn them about it beforehand. It is very necessary with enteric patients for the nurses always to be cheerful, and to take their patients a little into confidence. There are old patients of ours in England now who have fought a hard battle with life out here, yet are now strong and well.

### Royal British Nurses' Association.

The arrangements for the bazaar announced for December 6th, 7th, and 8th, at the Grafton Galleries, to be opened by Princess Christian, President of the Royal British Nurses' Association, were not sufficiently advanced on Wednesday night when we went to press to enable us to describe them fully. Her Royal Highnesr will personally superintend a stall, at which she will be assisted by her daughter, Princess Victoria of Schleswig-Holstein, and by Lady Jeune and Lady Duckworth. The General Hospitals' stall is under the charge of Miss Mollett, Miss Maud Smith, Miss Rogers, and Miss Bull. The Special Hospitals are represented by Miss Butler, Miss East, Miss Ridley, Miss Ross, Miss Elphick, Miss Cartwright, Miss Busby, Miss C. Bourne, Miss Court, and Miss F. Hole. The Middlesex Hospital stall is presided over by Miss Thorold, Mrs. Craufurd, Miss Temple Frere, and Miss G. Morgan. Miss Anderson takes charge of the Children's Hospital stall. The infirmaries' stall is in the hands of Miss F. M. Hughes, Miss Graham, Miss Elma Smith, Miss Wesley, and Miss Duffers. The Private Nursing stall will be managed by Mrs. Bedford Fenwick, Miss Beachcroft, and Miss Robertson. Miss Isla Stewart takes charge of the tea and refreshment room, and Miss De Pledge of the Dairy stall.

### From Two to Three Guineas.

"Nursing homes are so expensive" is the constant cry, and, therefore, the appearance of a new one where the charges are strictly moderate ought to be welcomed by persons of limited means. Two experienced nurses have opened a large and convenient house at 20, Endsleigh Gardens, Tavistock Square, W.C., where medical cases of all kinds can be received, the charge of separate rooms being from four guineas a week. A fine apartment, lofty and well-lighted, also furnishes ample space for two or even three patients, who can be comfortably boarded and nursed at from two to three guineas a week.

### Notes and Queries.

The contents of the Editor's Letter-box have now reached suchunwieldy proportions that it has become necessary to establish a hard and fast rule regarding Answers to Correspondents. In future, all questions requiring replies will continue to be answered in this column without any fee. If an answer is required by letter, a fee of half-a-crown must be enclosed with the note containing the enquiry. We are always pleased to help our numerous correspondents to the fullest extent, and we can trust them to sympathise in the overwhelming amount of writing which makes the new rules a necessity. Every communication must be accompanied by the writer's name and address, otherwise it will receive no attention.

### Queries.

(48) *Special Nursing.*—Please recommend a book on gynæcological nursing.

(49) *Nursing Abroad.*—Addresses of nursing institutes at San Remo and other foreign cities will be gratefully received.—*Nurse Lena.*

(50) *Lepers.*—Kindly give me address of Leper Hospital, South Africa. —*Nurse L.*

(51) *Monthly Nurse.*—Please let me know if you hear of any lady going to the Cape who wishes to take a monthly nurse with her.—*Nurse H. C.*

### Answers.

(48) *Special Nursing.*—"Notes on Gynæcological Nursing," by John Benjamin Hellier, M.D., M.R.C.S., price 1s. 3d., post free 1s. 5d. Also "Handbook of Obstetric and Gynæcological Nursing," by Dr. Fleetwood Churchill, post free 4s. 3d. These can be procured through The Scientific Press, 428, Strand, London.

(49) *Nursing Abroad (Nurse Lena).*—Miss Briant, 19, Via Vittorio Emanuele, San Remo; and Mrs. Norris, Les Agaves, Cannes. The superintendent of the Hollond Institute, Nice, can tell you of branches in different foreign towns.

(50) *Lepers (Nurse L.).*—You had better write to the Medical Superintendent or to the Matron at the Leper Hospital, Robben Island, Cape Town.

(51) *Monthly Nurse (Nurse H. C.).*—You had better advertise or answer advertisements.

### Everybody's Opinion.

[Correspondence on all subjects is invited, but we cannot in any way be responsible for the opinions expressed by our correspondents. No communications can be entertained if the name and address of the correspondent is not given, or unless one side of the paper only be written on.]

### NURSING IN SOUTH AFRICA.

"The Matron of the Albany General Hospital" writes: Although I cannot claim fifteen years' nursing experience, and am neither a member of the R.B.N.A. or of the R N.P.F., nevertheless I may be considered qualified to answer the communication written by "C. C. B.," seeing that I am an English-trained nurse (Nightingale Home and St. Thomas's and Charing Cross Hospitals), and that I have resided in this colony for nearly seven years. It will be best, perhaps, to consider seriatim the statements made by "C. C. B." (1) "For nurses wishing to obtain work in hospitals, and already trained in England, there is absolutely no opening." In all probability this statement may be made with truth in about five years' time, as there are now one or two colonial hospitals where nurses are receiving a good and thorough three years' training. At present, however, English-trained nurses find no difficulty in obtaining employment. There are several hospitals where only trained nurses are employed, and at present colonial-trained nurses have little chance in competing for a post against a home-trained nurse. The reason for this may partly be that up to recent years the colonists have been accustomed to receive all skilled work from English hands, because their own people had not had the opportunity of attaining to the same degree of proficiency, and now they are somewhat slow to recognise that they have among them quite as good material as they can import. When this prejudice wears off, as it will in a few years, then the English-trained nurse will not so readily find occupation in South Africa. (2) "The only large hospital in the colony is the New Somerset." "C. C. B." is evidently not aware that the Kimberley Hospital is considerably larger than the New Somerset. (3) As to the class from which the nurses are taken, "C. C. B." is again at fault. She has, it would seem, but very inadequate knowledge of "farmers" out here, or she would not speak of small storekeepers and farmers' daughters as though they necessarily belonged to the same class. I can only say that the number of lady nurses in the hospitals here is larger in proportion to the size of the whole nursing staff than it is in the London hospitals. (4) With regard to the nurses' salaries, they are certainly not high, but we all know that the nursing profession is one in which we have to give a great deal and receive very little of this world's goods in return. At the same time the salaries given in colonial hospitals are much higher than those given in English ones. At Kimberley they range from £20 to £60; at St. Thomas's, £12 to £50; and at Guy's, £8 to £30. (5) "All the other hospitals, with the exception of the Albany General Hospital in Grahamstown and the Port Elizabeth Hospital, are of the cottage class . . . miserable places, undernursed, badly constructed. . . The doctor, as a rule, young and inexperienced." At King Williamstown there is a very good Government hospital, the "Grey," which has an experienced resident doctor, a matron, four trained nurses, and one probationer. Queenstown, East London, Graafreinet, and one or two other smaller towns also have their own hospitals; small, certainly, but as a rule well-arranged and well cared for. (6) The servants. Are they not a universal grievance? "C. C. B.'s" assertion with regard to coloured labour is far too sweeping. There are good servants to be met with among the Kaffirs, who are many of them very clean in their habits and very capable workers. There is a great amount of drunkenness, dishonesty, and dirt, chiefly among the Hottentots and half-castes. (7) Private nursing. The description given by "C. C. B." of the horrors of private nursing may possibly be true of the Transvaal or Orange Free State, but in the colony the nurse will, as a rule, meet with great respect and gratitude. Often, indeed, only one nurse is available for a case which requires two, and there is a danger of too much being required of the one. This, however, will soon be remedied when the supply equals the demand. (8) "Women in the colony have no sense of decency. The tone, even among the best class, is frightfully low and sensual." This statement and other similar ones concerning the social and moral life of the colonists is false, and can only have been made by one who has had no acquaintance with what she calls the "average colonial," a term of which I fail to comprehend the meaning. (9) The climate. We should have thought that even if everything else in the colony were worthy of abuse, at all events the climate would have escaped censure. If "C. C. B," does not like this climate, what climate does she like? In conclusion, I will repeat what I said at the beginning, the colony will very soon be able to supply the demand for skilled nurses, and then there will no longer be any need for English ones to come out. But the colonists have yet to prove that they can hold their own in competing with the home-trained nurses. I am convinced that for more reasons than one a colonial nurse will be far better fitted for nursing in the colony than an English-trained nurse fresh from England. There is great interest in watching the gradual development of a new country like this, and it is with pleasure that one sees the colonists making efforts to supply their own needs, and if English people want to come out here to work, it should not be with a desire to supplant the colonists, but with the wish and the purpose to help them to develop their own material. For this reason I would, like the matron of the New Somerset, always choose my probationers from among the colonists.

### NURSING IN ASYLUMS.

"Five Years an Asylum Nurse" writes: In your Asylum News and Nursing, there was recently an account of a patient's escape, which may be misleading to many readers. In all large asylums no nurse is allowed to go out without a pass signed by medical superintendent or matron, consequently I imagine that the case referred to happened in a very small institution. I am aware that more medical and surgical training would be very beneficial to us (and gradually we are getting it), but everyone must acknowledge that the organization in our asylums is perfect, both with regard to patients and staff. For this reason I ask you kindly to insert this letter.

### Presentations.

A purse of gold was presented to Miss Bangham on her resignation of the post as Lady Superintendent of Moseley Hall Convalescent Home for Children, Birmingham, which she has held for nearly two years.

On December 1st Miss Pringle, who has been "Queen's Nurse" at Dysart, Fifeshire, for the last two and a half years, was presented by the Countess of Rosslyn, on behalf of the Nursing Committee, with a handsome gold watch-bracelet. Miss Pringle is going abroad to be married, and carries with her many kind wishes from her friends.

### Death in Our Ranks.

We hear with regret of the death of Mamie, wife of John Deans, M.D., at New Norfolk, Tasmania, on Oct. 20th. Mrs Deans (*née* Robinson) was well known and greatly esteemed at the Bristol General Hospital, where she was a valued nurse. "After life's fitful fever, she sleeps well."

### The Book World for Women and Nurses.

[We invite Correspondence, Criticism, Enquiries, and Notes on Books likely to interest Women and Nurses. Address, Editor, The Hospital (Nurses' Book World), 428, Strand, W.C.]

### MAGAZINES OF THE MONTH.

The Christmas number of the Pall Mall Magazine must surely offer attractions to all sorts and conditions of readers. A very large proportion of Her Majesty's loyal subjects ever receive joyfully any tidings of herself and other members of the Royal family. Such among us will be delighted with Mr. Beavan's contribution "Notable Portraits of the Queen and Royal Family." "The Rise of Wellington" by Lord Roberts, is continued, and will be most acceptable to the warlike spirits among us, and indeed to all patriotic Englishmen; whilst Mr. Besant's paper, entitled "Westminster," appeals to the artistic temperament, and to those who take an interest in ecclesiastical architecture. "Street Scenes in Cairo," by Mr. Hichens, is well worth reading. There are some good lines by Hamilton Aidé, and some humourous ones by J. Lionel Booth. No doubt Rider Haggard's story, "Joan Haste," now running through the numbers is a great attraction, and, as we know, is greedily devoured by his numerous admirers, whilst "Johnsson's Adventure," by Mr. Alden, is very amusing. Among other contributions to the magazine there is a charming story by Q., "The Bishop and Eucalyptus," full of pathos and beauty this is, pointing out as it does the gladdening truth, the power of one good unselfish life over much that is evil.

There is a variety of entertaining matter in the Ludgate Illustrated Magazine for December. Especially interesting just now, when the women are so much upon the *tapis*, is the article on "Girton College, Cambridge," the illustrations of which, from photographs, are good and very striking. A complete novel by Edwin Hughes entitled, "An Apostle of Freedom," deals with anarchists and bombs, so cannot fail to prove another up-to-date attraction. Tales of the service are always popular, and "Lancer and Dragoon," by Walter Wood, is powerfully told. It is a pity all separations do not end as quickly as that in Theodore Trippin's pretty little story, "A Deed of Separation." Other tales and articles make up the number. Amongst these we find "Fashion Notes," by Florence Mary Gardiner, and "Incidents of the Month," by John A. Steuart. Taken as a whole, this month's issue of the magazine is very good reading.

There are some excellent contributions to the Christmas number of The English Illustrated. That enterprising author, Mr. McKenzie, gives us an account of his experiences as a steerage passenger to New York, for the unprecedented sum of £1 16s. The author admits that he almost went back when he got on board, but changed his mind, we are glad to say. We catch glimpses of Shelley in Italy, and Professor Douglas contributes a paper on "Chinese Mandarins and People," which will be gratefully received at the present time. Clement Scott charms us with his reminiscences of Sir Edwin Arnold, and we long to visit Cornwall as we read "The Land of a Lost Language," by William Copeland Borlase. Some books of the year are reviewed by Mr. Austin, and there are a number of stories by popular authors. Helen Mathers goes into the vexed question of the New Woman in "Old versus New," and Mrs. Clifford touches on the same theme. "Two Mayors of Bottitort," by Mr. Stanley Weyman, is very amusing; so is "The Incomplete Highwayman," and "A Cut and a Kiss," by Anthony Hope. A weird little tale by Grant Allen and a pathetic one by Margaret Woods, entitled "Prison Bars," are both above the average in interest. "A Winter's Sport in the Rockies" will be appreciated by sportsmen. Several other tales complete the present issue of the magazine, perhaps the most charming of which is the first by Frances Hodgson Burnett, where little Betty's kitten tells her story.

Franco-English Review. Yearly subscription in England and France, 7f. 50c.; in other countries, 8f. 50c. (Published by Boyveau et Chevileet, 22, Rue de la Banque, Paris.)

The "Franco-English Review" of November 15th contains some excellent articles. One on Sir Joseph Barnby, with portraits of the musician himself and of his fair wife and daughter, is specially interesting. "Pictures by the Cam" is a brightly written sketch of life at Cambridge, evidently penned by a lover of the historic "Backs."

### Appointments.

Garrison Women's Hospital, Shorncliffe Camp.—Miss Frances Berry has been appointed Matron and Midwife at this hospital. She was trained at Westminster Hospital, where she worked in the wards and on the private staff for three years. Miss Berry obtained her midwifery experience at the Garrison Women's Hospital, Woolwich, and holds the L.O.S.

Rotherham Hospital and Dispensary.—Miss Julia Sanders has been appointed Matron of the Rotherham Hospital and Dispensary. She was trained at St. Marylebone Infirmary, where she has held the post of Sister for several years, and done much credit to her training. We hope she may soon make as many friends at Rotherham as she leaves at Marylebone.

Royal Edinburgh Hospital for Sick Children.—Miss E. F. Piggott has been appointed Matron of this hospital.

Stratford-on-Avon Joint Fever Hospital.—Miss Alice Taylor has been appointed Matron of this hospital. She held a similar post at the Cambridge Infectious Hospital for over two and a-half years, and has excellent testimonials for nursing and administration, being also a thoroughly good housekeeper. We wish her continued success in her new work.

Tewkesbury Hospital.—Miss Blanche Mays has been appointed Matron of the Tewkesbury Hospital. Miss Mays was trained at the Liverpool Northern Hospital; for the last two years she has held the post of Sister at St. Marylebone Infirmary, where she has done excellent work. We wish her every success in her new post.

Victoria Hospital, Folkestone.—The post of Matron at this hospital has been conferred upon Miss J. Barbar, who was trained in the Nightingale School, St. Thomas' Hospital, where she afterwards took Sister's duties in various wards, and was in temporary charge of the infectious block. Miss Barbar was First Assistant Matron at the County Asylum, Chatham. We congratulate her on her promotion.

Victoria Hospital, Burnley.—Miss C. R. Rigney has been appointed matron of this hospital. She was trained at the Liverpool Royal Infirmary, and was subsequently charge nurse, night superintendent, and Assistant Lady Superintendent in the same institution. Miss Rigney's testimonials are excellent, and we congratulate her on her appointment.

### Minor Appointment.

Victoria Cottage Hospital, Kington.—Miss J. M. Johnson has been appointed nurse matron of this hospital. She was trained at the General Hospital, Birmingham, and afterwards held posts in the Huddersfield Infirmary; Dewsbury and District General Infirmary; Rugby Hospital; Eaton Hospital; Middlesborough and Rochdale Infirmary. We wish Miss Johnson every success.

### Wants and Workers.

Will some one give a musical box to the patients in a union in Wales? The sick have no visitors, and but very little amusement of any kind, and would appreciate the suggested gift.—*Nurse R. S.*

Can anyone recommend a home near London where a chronic case of rheumatism could be received on payment of £20 per annum? Patient is 70 years of age, formerly a governess; is the daughter of a solicitor. This small income precludes her entering many institutions.—*Norwood.*

Admittance to a Convalescent Home (free) is urgently needed for a poor man with a large family, who requires a change to bracing air before he can be fit to work again.—*Essex.*

## Figures and Tables

**Figure f1:**